# Conductive and Thermo-Responsive Composite Hydrogels with Poly(N-isopropylacrylamide) and Carbon Nanotubes Fabricated by Two-Step Photopolymerization

**DOI:** 10.3390/polym15041022

**Published:** 2023-02-18

**Authors:** Gianluca Ciarleglio, Elisa Toto, Maria Gabriella Santonicola

**Affiliations:** Department of Chemical Engineering Materials Environment, Sapienza University of Rome, Via del Castro Laurenziano 7, 00161 Rome, Italy

**Keywords:** poly(N-isopropylacrylamide), hydrogels, carbon nanotubes, photopolymerization, calorimetry

## Abstract

Biocompatible and conductive polymer hydrogels are the subject of intensive research in the bioengineering field because of their use in bioelectronic devices and for the fabrication of electro-responsive tissues and drug delivery systems. In this study, we report the synthesis of conductive composite hydrogels consisting of a poly(N-isopropylacrylamide) (PNIPAM) matrix embedding carboxyl-functionalized multi-walled carbon nanotubes (MWCNT-COOH) using a two-step photopolymerization method. Thermo-responsive hydrogels with controlled hydrophilicity and conductivity were prepared by varying the carbon nanotube concentration in the range 0.5–3 wt%. The thermal response of the PNIPAM-based composite hydrogels was measured by differential scanning calorimetry with both ultrapure water and PBS solution as swelling liquid. Results show that the endothermic peak associated with the temperature-induced volume phase transition (VPT) shifts to higher temperatures upon increasing the concentration of the nanotubes, indicating that more energy is required to dissociate the hydrogen bonds of the polymer/filler network. In PBS solution, the swelling ratios and the VPT temperatures of the composite hydrogels are reduced because of salt-induced screening of the oppositely charged polymer/filler assembly, and the electrical resistivity decreases by a factor of 10 with respect to the water-swollen hydrogels.

## 1. Introduction

Hydrogels are three-dimensional (3D) networks of cross-linked polymers capable of absorbing and retaining large amounts of water [1,2]. In the biomedical field, biocompatible and electrically conductive hydrogels are currently used for several applications, such as biosensing, drug delivery, wound dressings, and tissue engineering (TE) [3,4,5]. In particular, hydrogels play an increasingly important role and are extensively researched in tissue engineering due to their unique properties, including high permeability, controlled biodegradation, and ability to provide support to engineered tissue structures [6,7]. In addition, they possess tunable physical properties and can be chemically modified to facilitate the bioconjugation of active molecules [8,9]. In tissue engineering, various injectable hydrogels, designed with a broad range of mechanical properties, have been employed to rebuild myocardial function [10,11,12,13,14]. After myocardial injury, the fibrotic tissue [15] that forms is not mechanically and conductively functional [16,17] and leads to myocardial remodeling and eventual heart failure. Tissue engineering provides a new approach for restoring myocardial function using scaffolds that restore myocardial conductivity and the mechanical function of the heart.

For structurally challenging TE applications, the use of hydrogels is limited by their poor mechanical properties and composite hydrogels with integrated fillers are often used to prepare materials with enhanced mechanical properties. Various nanomaterials, including carbon-based nanoparticles (carbon nanotubes [18], graphene), metal [19], polymeric [20], and silica [21] nanoparticles, have been incorporated into polymer networks to form composite hydrogels. Among them, carbon-based nanomaterials and metal nanoparticles are widely studied to obtain electrically conductive hydrogels. In general, these hydrogels find applications in tissue regeneration, particularly in nervous or cardiac applications where electrical conductivity is crucial for the effective functionality of the native tissue [22,23,24]. Shin et al. [25] engineered composite hydrogels based on methacrylate gelatin (GelMA) incorporated with reduced graphene oxide (rGO), which conferred electrical conductivity and good mechanical properties to the hydrogel. In addition, cells cultured on rGO-GelMA composite scaffolds showed improved biological activities. Jo et al. [26] developed graphene hydrogels by mild chemical reduction of graphene oxide/polyacrylamide (GO/PAAm) with high conductivity and tissue-like mechanical properties having good compatibility in vitro and in vivo. The application of electrical stimuli through the conductive graphene hydrogels revealed a significant increase in the myogenic gene expression levels of myoblasts. However, the use of GO and rGO for the fabrication of electrically conductive hydrogels has some disadvantages. In particular, the electrical conductivity of GO and rGO cannot compete with that of carbon nanotubes (CNTs), and the oxidation and reduction reactions of graphene are elaborate and time-consuming [27].

Regarding CNTs, they have shown multiple functionalities, which have been utilized in biological systems [28,29] and integrated in hydrogels. For instance, Shin et al. [30] fabricated methacrylate gelatin (GelMA) cardiac patches containing CNTs and showed that GelMA hydrogels significantly improved electrophysiological and mechanical properties with the addition of MWCNTs. They varied the concentrations of CNTs to adjust the mechanical modulus and the electrical conductivity of these hydrogels. However, in this case, non-functionalized carbon nanotubes were used. This may be limiting, since the addition of functional groups on the nanotube surface has been shown to enhance the polymer–filler interactions and this the performances of the resulting composites [31]. Flexible hybrid hydrogels with high electrical conductivity, thermal responsiveness, and pH-sensitivity were developed by Zhan et al. [32]. In this case, the process of in situ polymerization was used to prepare MWCNT-reinforced PNIPAM/carboxymethyl chitosan (CMCS) hydrogels, yet the water content and swelling behavior of these materials were not considered, although they are of great interest for biomedical applications.

Poly(N-isopropylacrylamide) (PNIPAM) is a thermosensitive polymer composed of both hydrophilic amide groups (-CONH-) and hydrophobic isopropyl side chains (-CH(CH_3_)_2_) [33,34]. PNIPAM has a lower critical solution temperature (LCST) of approximately 32 °C, which is lower than the body physiological temperature [35,36]. Moreover, PNIPAM solutions assume a sol state at room temperature and can be transformed into more viscous gels by increasing their temperature to 37 °C [37]. For these reasons, PNIPAM has been used in a wide range of biomedical applications, including cell therapy [38,39], drug delivery [40], for scaffolds in tissue engineering, and for fabricating antibacterial surfaces [41,42]. For instance, Cunliffe et al. tested the attachment of *Salmonella typhimurium* and *Bacillus cereus* for PNIPAM brushes, demonstrating that bioadhesion at surfaces can be controlled by modulating the environment temperature. In particular, both types of bacteria showed a strong adhesion to PNIPAM coatings at temperatures above the LCST and weak adhesion below the LCST [42].

PNIPAM-based injectable hydrogels [43,44] have been extensively employed as cell carriers for in vivo tissue engineering. Wang et al. [45] developed a family of highly flexible hydrogel composites based on collagen, chondroitin sulfate and a thermosensitive and degradable PNIPAM copolymer. These hydrogel composites showed good mechanical properties, oxygen permeability, and were also able to release bioactive IGF-1 during a two-week period. They can be used as transporters of growth factors and therapeutic cells for cardiovascular tissue engineering. Other PNIPAM-based injectable hydrogels have been developed for tissue repair. For example, Atoufi et al. [46] synthesized thermosensitive injectable hydrogels based on PNIPAM and hyaluronic acid containing varying amounts of chitosan-acrylic acid-coated PLGA micro/nanoparticles designed to facilitate cartilage tissue regeneration.

In this work, the fabrication of PNIPAM-based hydrogels containing carboxyl-functionalized multi-walled carbon nanotubes (MWCNT-COOH), to make the hydrogels electrically conductive, is investigated. A two-step photopolymerization approach was developed to effectively incorporate the MWNCT-COOH in the PNIPAM matrix and to obtain a homogeneous composite hydrogel. This procedure allows to incorporate carbon nanotubes within the polymer matrix using a biocompatible and cell-viable agent, the photoinitiator Irgacure 2959 [47,48,49], avoiding the radical polymerization approach which involves toxic reagents, such as N,N,N′,N′-tetramethyl ethylenediamine (TEMED) and ammonium persulfate (APS) [50,51,52]. The stable integration of carbon nanotubes with the polymer matrix is a critical step as free nanomaterials might bring toxic effects in biological systems, whereas they have been shown to be cytocompatibile when incorporated into polymer matrices [18,53]. The swelling and thermo-responsive properties of the PNIPAM/MWCNT-COOH hydrogels are analyzed, and the volumetric phase transition (VPT) determined. A surface wettability analysis is performed using the contact angle method, whereas degradation tests in water are used to assess the potential tendency of the composite hydrogels to release the MWCNT-COOH filler. Finally, the conductivity of the hydrogels is investigated by electrical impedance spectroscopy (EIS).

## 2. Materials and Methods

### 2.1. Materials

Carboxyl-functionalized multi-walled carbon nanotubes (MWCNT-COOH) with average outer diameter 30 nm and length 1–5 µm (PD30L1-5-COOH, purity > 95%) were obtained from NanoLab (Waltham, MA, USA). These nanotubes were prepared by reflux in concentrated sulfuric/nitric acid and are highly functionalized (2–7 wt% of COOH groups by titration as reported by the manufacturer). The following products were supplied by Sigma-Aldrich (Schnelldorf, Germany) and used without further purification: N-Isopropylacrylamide (NIPAM, 97%, 415324), N,N′-methylenebisacrylamide (MBA, 99%, 146072), and 2-hydroxy-4′-(2-hydroxyethoxy)-2-methylpropiophenone (Irgacure 2959, 98%, 410896). Ultrapure deionized water (resistivity 18.2 MΩ·cm) was produced by a Direct-Q3 UV water purification system (Millipore, Molsheim, France) and used in all preparations.

### 2.2. Hydrogel Synthesis

The PNIPAM/MWCNT–COOH hydrogels were prepared using a two-step photopolymerization method. First, NIPAM monomer (0.1 g/mL) and Irgacure 2959 photoinitiator (0.01 g/mL) were added to ultrapure water and mixed for 30 min in cold water bath using a magnetic stirrer (C-MAG HS7, IKA, Staufen, Germany). The solution was then refrigerated at 4 °C for 24 h in the dark. Next, 3-mL aliquots of the NIPAM/Irgacure solution were transferred to Petri dishes and photopolymerized under UV-A light (λ 365 nm, flux 2.3 mW/cm^2^) for 15 min to obtain a homogeneous gel of poly(N-isopropylacrylamide) (PNIPAM). In the second step, the carbon nanotubes were added to the PNIPAM gel at different concentrations (0.5%, 1%, 2% and 3% by weight with respect to the initial NIPAM content). The dispersions were sonicated for 15 min in ultrasonic bath (Elmasonic P30H, Elma, Singen, Germany) filled with cold water to prevent heating of the thermo-responsive gel, followed by magnetic stirring for further 15 min. Next, the crosslinking agent MBA and Irgacure 2959 were added to the dispersions using a ratio of NIPAM:MBA:Irgacure 2959 of 10:3:3 by weight. The mixtures were stirred in cold water bath for 30 min, stored at 4 °C for 24 h in the dark, and then immediately photopolymerized under UV-A light for 1 h. After the second step of photopolymerization, the hydrogels were washed by immersion in ultrapure water (5 cycles over 3 days) to remove unreacted monomers and reagents.

A Leica DMLP polarized microscope equipped with 5×, 10×, and 20× objective lenses was used to analyze the dispersion of the carbon nanotubes in the PNIPAM gel. The MWCNT-COOHs were dispersed in the gel by sonication at frequency 37 kHz for 15 min. For imaging, the mixtures were spread onto glass microscope slides (Menzel-Gläser) and covered with coverslips to prevent water evaporation.

The yield of the two-step reaction was determined for each type of the composite hydrogel using the following equation:

(1) $$\mathrm{Yield}\;\left(\%\right)=\frac{W_{d}}{\sum_{i}W_{i}}\times 100$$

where W_d_ and W_i_ are the weight of the dried hydrogel and the weight of the initial monomer and crosslinker in solution, respectively. To determine W_d_, the hydrogel samples (five replicates for each type of hydrogel) were dried at 50 °C for 24 h and immediately weighed to prevent them from absorbing moisture.

### 2.3. Hydrogel Swelling and Water Content Measurement

The dried hydrogels were immersed in deionized water or in phosphate-buffered saline (PBS) solution for 2 days at room temperature (T = 25 °C). Next, the swollen hydrogels were removed from solution, blotted with a tissue paper to remove excess water on the surface, and then weighed until a constant weight was obtained. The swelling ratio and the content of the water were calculated as follows [54]:

Swelling ratio = (W_s_/W_d_) (2)

% Water content = ((W_s_ − W_d_)/W_s_) × 100 (3)

where W_s_ is the weight of the hydrogel in its equilibrium swollen state and W_d_ the weight of the completely dried hydrogel.

### 2.4. Degradation Tests

Degradation tests were performed on 12 samples of PNIPAM/MWCNT-COOH hydrogel containing 3 wt% of carbon nanotubes. Each sample (350 mg) was immersed in PBS solution (25 mL) and kept at 40 °C. The degradation was assessed over 4 weeks by examining the presence of hydrogel material in the supernatants. After each week, 3 samples were analyzed and compared with a reference PBS sample that had not been in contact with hydrogel. The degradation was evaluated by weighing the residue in the supernatants above the immersed hydrogels over the time.

### 2.5. Differential Scanning Calorimetry

The glass transition temperature (*T_g_*) and the volume phase transition (VPT) of the composite hydrogels were investigated using differential scanning calorimetry (DSC). A Perkin Elmer DSC 8500 instrument (Perkin-Elmer, Waltham, MA, USA) calibrated with high purity indium and tin was used. DSC samples were sealed in aluminum pans with lids and measurements were performed under a constant flow of nitrogen (20 mL/min). An Identical empty cell was taken as reference. For the determination of *T_g_*, dried hydrogel samples (5 samples, ~10 mg) were analyzed in the temperature range of 20–250 °C at a heating rate of 5 °C/min. For the determination of the VPT peaks, swollen hydrogel samples (~25 mg) were measured in the temperature range 20–50 °C at a heating rate of 10 °C/min. Samples were fully equilibrated in deionized water or PBS solution for 48 h before DSC measurements. DSC thermograms were analyzed using the Perkin Elmer thermal analysis software provided with the instrument. The determination of *T_g_* was performed according to ASTM Standard E1356-08 [55].

### 2.6. Contact Angle Measurements

Contact angle measurements were performed to compare the degree of hydrophilicity of the synthesized hydrogels. Contact angles were measured at room temperature by the sessile drop method using a video-based optical analyzer (OCA 15 Pro, DataPhysics Instruments, Filderstadt, Germany) equipped with an automatic singe dosing system for micro-syringe and a high-speed video camera. Droplets (volume 3 µL) of deionized and degassed water were used for the measurements. Droplet spreading at the surface of the fully hydrated hydrogel samples was recorded for several seconds and analyzed using the SCA 20 image analysis software (DataPhysics Instruments, Filderstadt, Germany) provided with the instrument.

### 2.7. Electrical Impedance Spectroscopy

Electrical impedance measurements were carried out on composite hydrogel samples using a Reference 600 potentiostat instrument (Gamry Instruments, Warminster, PA, USA) with a four-electrode configuration setup. Prior to measurements (10 replicates for each sample type), the PNIPAM/MWCNT-COOH hydrogels at filler concentration of 1, 2, and 3 wt% were fully equilibrated in deionized water or in PBS at room temperature. Electrical impedance spectroscopy (EIS) was conducted in the frequency range 0.1 Hz–500 kHz. The VistaShield Faraday cage (Gamry Instruments) was used to remove ambient electronic noise that could negatively affect measurements. A sine signal with amplitude of 10 mV was used as source signal. Hydrogel samples were placed in a square measurement cell (5 cm × 5 cm) made of Teflon, containing two copper electrodes (1 cm × 1 cm) at the bottom and upper side in direct contact with the sample. A 1-mm thick polydimethylsiloxane (Sylgard 184) spacer with a square 1 cm × 1 cm window in the middle was used to contain the hydrogel sample and to prevent its collapse between the electrodes. Impedance data were fitted by equivalent circuit models using the Gamry Echem Analyst software v. 7.07 provided with the instrument.

## 3. Results and Discussion

### 3.1. Composite Hydrogels by Two-Step Photopolymerization

**Figure 1:**
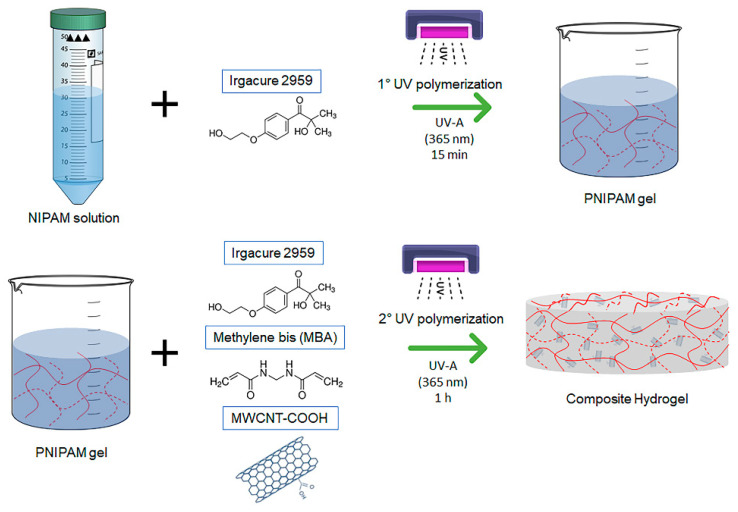
Scheme of the synthesis of PNIPAM/MWCNT-COOH hydrogel by two-step photopolymerization under UV light.

The synthesis of the PNIPAM/MWCNT-COOH composite hydrogels was conducted using a two-step photopolymerization approach. This procedure made it possible to successfully integrate the carbon nanotubes within the polymer matrix with a good degree of dispersion. On the contrary, previous preliminary syntheses, carried out following a single-step procedure, resulted in hydrogel samples characterized by a highly inhomogeneous distribution of the filler. Figure 1 shows a schematic representation of the procedure. The first step involved the synthesis of a PNIPAM gel starting from an aqueous solution of NIPAM monomer and water-soluble photoinitiator Irgacure 2959 that was polymerized under UV-A light. The photopolymerization was conducted for 15 min and resulted in a clear and homogeneous gel. In the second step, carboxyl-functionalized MWCNTs were added to the PNIPAM gel and immobilized in the polymer network via a second photopolymerization cycle lasting 1 h under UV-A light. To the best of our knowledge, the fabrication of this type of composite hydrogel, using in situ photopolymerization under UV-A light and mediated by cell-compatible Irgacure 2959, is a novel approach, which carries great potential for biomedical applications. This method could be particularly advantageous in tissue engineering since it can be carried out in the presence of living cells.

Different concentrations of carbon nanotubes ranging from 0.5 to 3 wt% were used in the process to fabricate hydrogels with varying electrical properties. MWCNT-COOHs were dispersed in the PNIPAM gel obtained after the first photopolymerization using ultrasound sonication in cold water bath. This method was proven to be effective to disperse the nanotubes in the polymer gel (Figure S3). The mechanical agitation resulting from compression zones of ultrasonic waves causes shear forces that can breakdown the strong van der Waals forces aggregating the nanotubes. In addition, due to the presence of the multiple carboxyl groups at its surface, the MWCNT-COOH filler showed good dispersion in the water-based PNIPAM gel, which is highly hydrophilic at temperatures below its critical phase transition.

**Figure 2:**
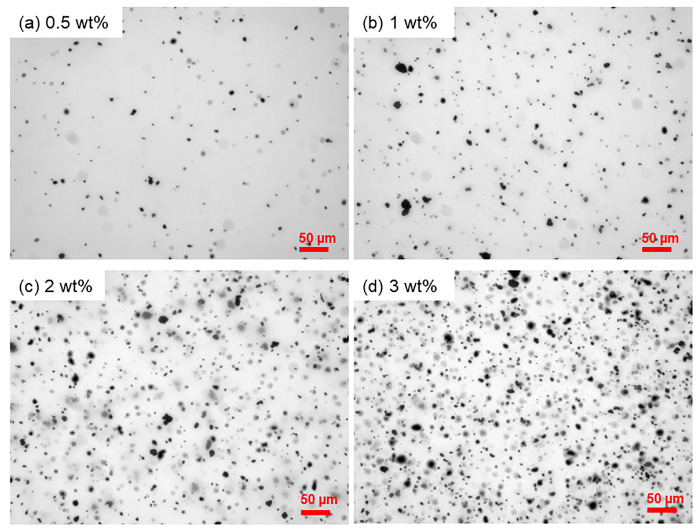
Optical microscopy images (20× objective) of MWCNT-COOH dispersion in the PNIPAM gel obtained by the first photopolymerization step. Concentration of nanotubes equal to (**a**) 0.5 wt%, (**b**) 1 wt%, (**c**) 2 wt%, (**d**) 3 wt%.

Figure 2 shows the dispersion of the MWCNT-COOHs in the PNIPAM gel, at different filler concentrations, after sonication for 15 min in cold water bath. Nanotubes functionalized with the carboxyl group form micro-sized aggregates, with mean particle size in the range of 4–6 μm (Figure S1 and Table S1), which appear homogeneously dispersed in the polymer matrix without the formation of macro-entanglements. In the case of the PNIPAM gel, the dispersion of the nanotubes is facilitated by electrostatic interactions between the negatively charged carboxyl groups at the nanotube surface and the positively charged amino groups on the macromolecules in the proximity of neutral pH conditions.

**Figure 3:**
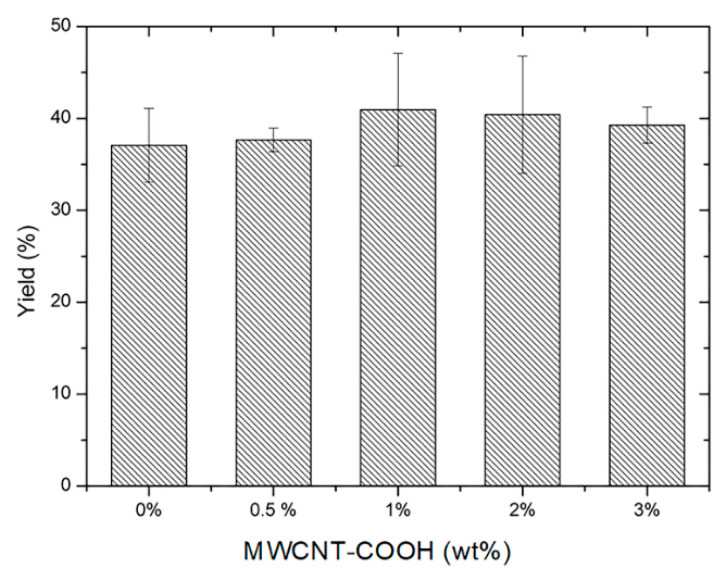
Reaction yield for the PNIPAM/MWCNT-COOH hydrogel synthesis with different nanotube concentrations.

The reaction yield of the two-step photopolymerization leading to the composite hydrogels was evaluated through Equation (1) and plotted as a function of the nanotube filler concentration (Figure 3). The yield is within the range of 37–41%, and there is no statistically significant difference upon an increase of the MWCNT-COOH concentration in the PNIPAM gel.

### 3.2. Hydrogel Swelling Behavior

**Figure 4:**
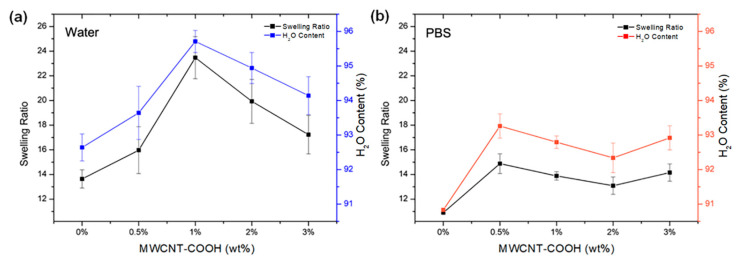
Swelling ratio and water content of PNIPAM/MWCNT-COOH hydrogels as a function of nanotube concentration in (**a**) pure water and (**b**) PBS solution at 25 °C.

The swelling capacity of hydrogels in aqueous media is strictly related to the presence of hydrophilic functional groups, such as hydrogen-bonding hydroxyl and carboxyl groups, on the polymer chains. These groups bind and retain water molecules, diffusing into the hydrogel network by capillary action and osmotic pressure difference. Figure 4 shows the swelling ratio and water content of PNIPAM/MWCNT-COOH hydrogels with different concentrations of carbon nanotubes after being completely equilibrated in pure water and in PBS at room temperature. Measurements were conducted at T = 25 °C, below the temperature of the volume phase transition of the hydrogels. A peak at 1 wt% of MWCNT-COOH is clearly visible for the composite hydrogels swollen in water, whereas in PBS solution maximum values of both swelling ratio and water content are anticipated at approximately 0.5 wt%. At higher concentrations of MWCNT-COOH, the swelling behavior in PBS is almost the same as the variations are within statistical errors. In general, the swelling capacity of hydrogels in PBS is lower than the degree of swelling of samples equilibrated in pure water. This result can be attributed to a charge-shielding effect caused by the presence of the additional salt ions in the PBS solution, which reduces the ionic electrostatic repulsion in the polymer network. Furthermore, in PBS, the osmotic pressure, resulting from the difference in the concentration of mobile ions between the gel and the aqueous phase, decreases and so does the amount of solution that is absorbed by the material.

It is noted that the water content of the PNIPAM/MWCNT-COOH hydrogels at 25 °C, corresponding to the swollen state of the hydrogels below their volume phase transition, is exceptionally high, reaching values above 90% in both cases, in pure water and in PBS solution. The strong hydrophilicity of the PNIPAM-based hydrogels at room temperature is related to the temperature-dependent interaction of the polymer amide groups with water. In the case of PNIPAM, which exhibits a coil-globule transition at temperatures around 32 °C [56], hydrogen bonding between the amide groups and water is dominant at temperatures below the critical solution temperature, whereas at higher temperatures hydrogen bonds among the amide groups of the polymer chains become predominant causing shrinking of the PNIPAM hydrogels [57]. The swelling ratio and water content of the composite hydrogels with carboxylated nanotubes are generally higher than those of the pure PNIPAM hydrogels and show a peak as a function of the filler concentration. This behavior highlights the important role that nanotubes with carboxyl groups have on the swelling behavior of the PNIPAM hydrogels. Indeed, the MWCNT-COOH used in this work are highly hydrophilic, carrying a large concentration of carboxyl groups, up to 7 wt% by titration as reported by the manufacturer. The large content of hydrogen-bonding groups on these nanotubes was previously confirmed by surface-enhanced Raman spectroscopy using SERS-active nanostructured substrates [58]. Increasing the MWCNT-COOH concentration enhances the swelling of the composite hydrogels up to a threshold value, after which this effect is reduced likely due to nanotube aggregation. In water, the peak of the swelling ratio is more pronounced (23.5 ± 1.7) and observed at 1 wt% of MWCNT-COOH (Figure 4a), whereas it is weaker (14.9 ± 0.8) and anticipated at around 0.5 wt% in PBS (Figure 4b). In PBS solution, the high ionic strength limits the swelling by screening the repulsive interaction among the ionized groups. This phenomenon also causes MWCNT-COOH aggregation at lower concentrations, thus shifting the swelling peak below 1 wt%.

### 3.3. Degradation Studies of Composite Hydrogels

Degradation studies were conducted over the time of four weeks to evaluate the possible release of MWCNT-COOH due to the degradation of the PNIPAM matrix of the composite hydrogels. Data related to the material residues in the supernatant above the hydrogels, while immersed in PBS solution at room temperature, were collected over four weeks and they are summarized in Table 1. A slight increase in the residue was observed over the weeks, with values of 0.20% after two weeks and 0.30% after one month. This study confirmed the overall stability of the composite hydrogels in PBS with negligible degradation of the polymer. The supernatant was analyzed under optical microscope and found to be free from residues after repeated centrifugation. The high stability of the composite hydrogels in PBS at physiological pH is due to the strong interactions between the protonated amine groups of PNIPAM and the deprotonated carboxyl groups of the nanotubes. In buffer solutions with acidic pH, a possible release of the nanotube filler in the surrounding environment, with loss of the hydrogel electrical properties and possibly toxic effects, should be considered. This would be a consequence of the larger number of carboxyl groups that are in neutral state, leading to a decrease of the interactions between the nanotubes and the PNIPAM network.

### 3.4. Calorimetry Analysis

**Figure 5:**
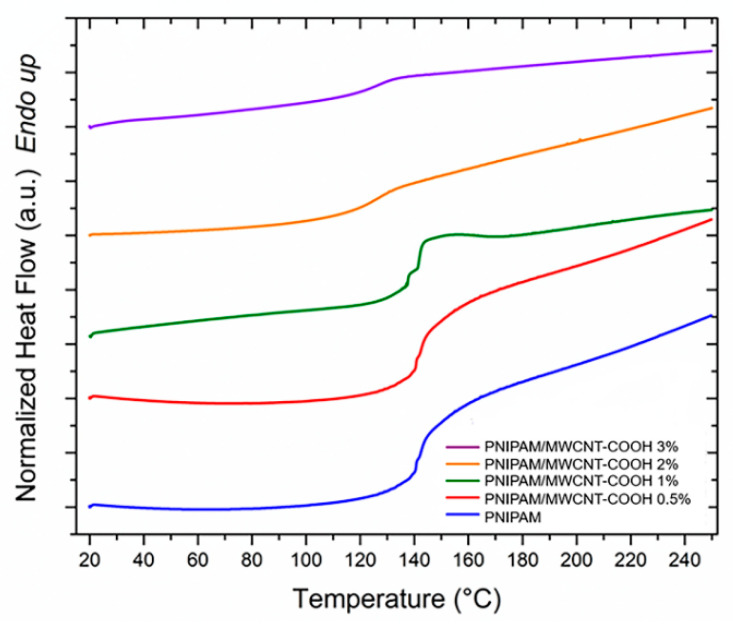
DSC curves (heating rate 5 °C/min) for *T_g_* evaluation of PNIPAM/MWCNT-COOH hydrogel samples at different concentrations of carbon nanotubes.

**Figure 6:**
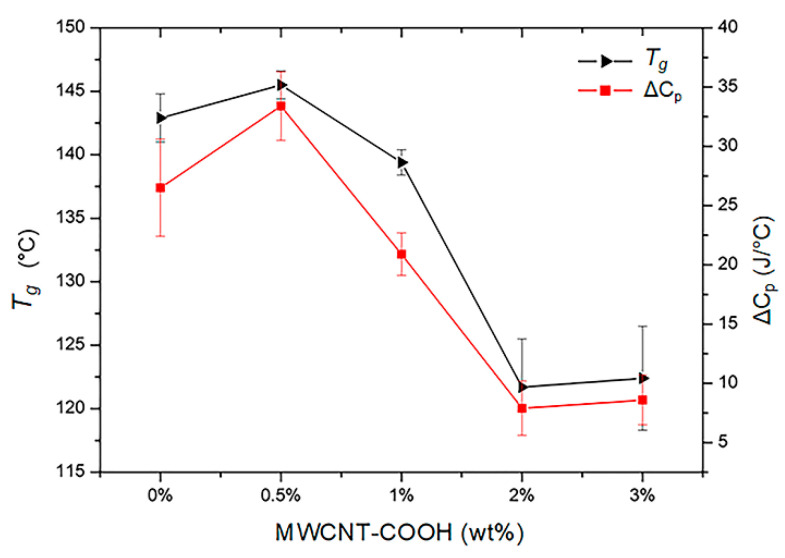
*T_g_* and ∆C_p_ trends of PNIPAM/MWCNT-COOH hydrogels as a function of filler concentration.

Differential scanning calorimetry was used to analyze the glass transition temperature (*T_g_*) and the volumetric phase transitions, induced by temperature variations, of the composite hydrogels with PNIPAM-based matrix and different concentrations of MWCNT-COOH nanotubes. For the evaluation of the *T_g_*, the hydrogels were completely dried, and five different samples were measured for each hydrogel type. Figure 5 shows typical DSC thermograms (range 20–250 °C) obtained from the dried hydrogels at different filler concentration. There is a slight increase of *T_g_* at low filler concentrations, followed by a marked decrease at higher concentrations of MWCNT-COOHs within the polymer matrix (Figure 6). Putz et al. [59] have suggested that such a decrease can be explained by two possible phenomena leading to an effective decrease in the crosslinking density of the hydrogels: network disruption near the polymer-nanotube interfaces or phase segregation. In this work, the MWCNT-COOH nanotubes represent a steric hindrance leading to a decrease in the network crosslinking density, and therefore a decrease in *T_g_*, when the filler concentration is increased. A decrease in the thermal capacity of the composite hydrogels is also observed as the concentration of nanotubes increases (Figure 6). Studies indicate how this decrease suggests a favorable interaction of the polymer with nano-sized fillers [60].

**Figure 7:**
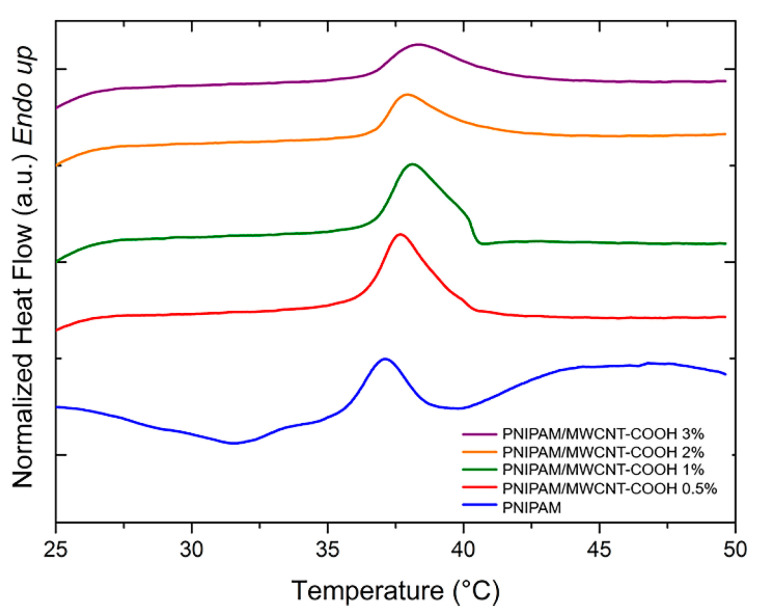
DSC curves (heating rate 10 °C/min) for determination of volume phase transition peak of PNIPAM/MWCNT-COOH hydrogels at different filler concentrations hydrated in pure water.

**Figure 8:**
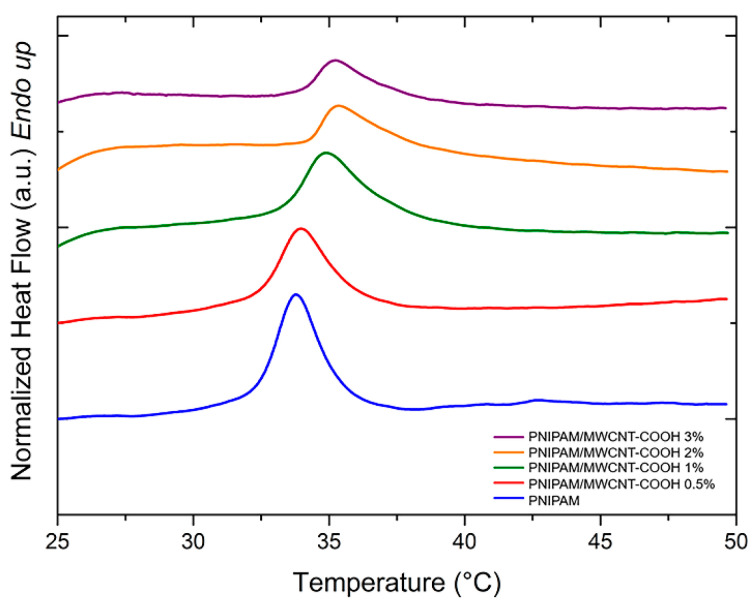
DSC curves (heating rate 10 °C/min) for determination of volume phase transition peak of PNIPAM/MWCNT-COOH hydrogels at different filler concentrations hydrated in PBS solution.

PNIPAM-based hydrogels are temperature sensitive, and they exhibit a volume change upon variation of the temperature, which is a phase separation process accompanied by an endothermic peak evolution. Figure 7 and Figure 8 show the DSC curves of PNIPAM/MWCNT-COOH hydrogels after equilibration at room temperature in pure water and in PBS solution, respectively, for the determination of the volume phase transition (VPT) peaks. In general, unfilled PNIPAM hydrogels show lower peaks and onset temperatures with respect to hydrogels with carboxyl-functionalized nanotubes.

The peak temperatures of the VPT and the corresponding enthalpy variations (ΔH) of the hydrogels swollen in pure water and in PBS solution are summarized in Table 2. In both cases, the composite hydrogels show a steady increase of the VPT temperature as a function of the MWCNT-COOH concentration. This result might be due to the complexation effect of the nanotubes with the polymer network, which causes an increase of the energy needed for the volume phase transition to occur. In addition, due to the carboxyl functionalization of the nanotubes, the composite hydrogels may form more hydrogen bonds with water. Consequently, more energy is needed to break these bonds thus shifting the transition peak to higher temperatures. The experiments conducted in PBS solution showed that the presence of salt ions lowered the transition temperatures by a few degrees when compared to tests conducted in deionized water. In PBS, the presence of kosmotropic agents, such as the phosphate anions, causes a reduction of the water–PNIPAM interactions [61]. In these conditions, the phase transition of PNIPAM resolves in two steps: the first is related to the dehydration of the amide group, while in the second there is a loss of the water molecules that hydrate the polymer [61,62]. Therefore, the reduction of water–polymer interactions results in a lowering of the VPT temperatures in PBS.

The intensity of the temperature-induced volume phase transition of the PNIPAM hydrogels is influenced by the presence of the MWCNT-COOH filler. This effect can be quantified by the enthalpy variation (ΔH) associated with the endothermic transition. For the composite hydrogels, a steady decrease of ΔH with the nanotube concentration is observed above 1 wt% for the hydrogels in water and above 0.5 wt% for those in PBS (Table 2). This result indicates that the hydrogel shrinking process is hindered by the presence of the nanotube network, which binds to the polymer matrix, thus limiting its collapse.

### 3.5. Surface Hydrophilicity of PNIPAM/MWCNT-COOH Hydrogels

**Figure 9:**
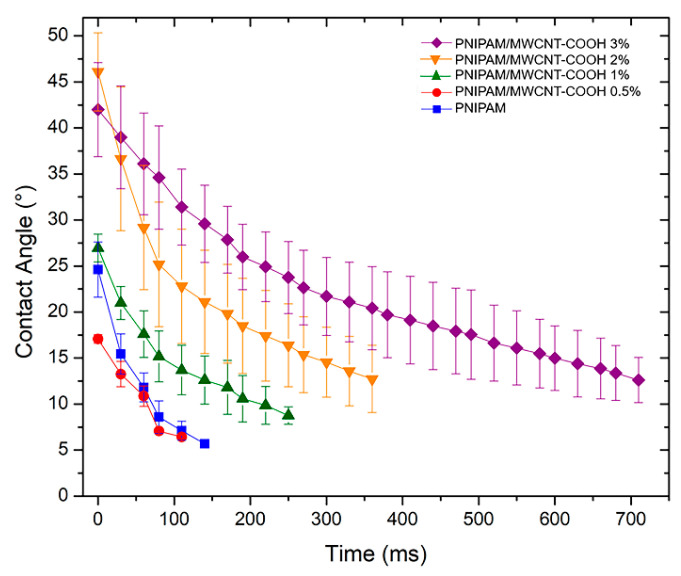
Time evolution of water contact angles at the surface of PNIPAM/MWCNT-COOH hydrogels with different concentrations of the carbon nanotube filler. Data measured at room temperature by optical tensiometry with values averaged over 10 replicates for each hydrogel type.

Contact angle (CA) measurements with water as testing liquid were performed to determine the hydrophilicity degree at the surface of the PNIPAM hydrogels with and without carbon nanotubes. These experiments were conducted at room temperature (25 °C), i.e., in the hydrophilic state of the hydrogels below their volume phase transition. Water droplets were deposited on each sample surface and their evolution in time was recorded. Figure 9 shows the average values of the water contact angles for the pure and composite hydrogels, previously equilibrated in water at room temperature. Representative images of the contact angle analysis at three different times (initial, half, and end) of the spreading are shown in Figure S2. Water droplets quickly spread over the surfaces within the time of 1 s at most, indicating the high degree of hydrophilicity of the PNIPAM-based hydrogels. The hydrophilicity does not vary significantly at low concentrations of MWCNT-COOH (up to 0.5 wt%), whereas the CA values steadily increase at 1 wt% of filler and above. For the composite hydrogels with 3 wt% of MWCNT-COOH, the initial value of CA is above 40° and it decreases to 10° in about 1 s, indicating a more hydrophobic nature of the composite hydrogel due to the presence of the carbon filler. In addition, from the results in Figure 9, it is noted that the slope of the kinetic curves varies with the type of hydrogel sample, being less steep at higher nanotube concentrations. This behavior may be due to an increase of the surface roughness caused by nanotubes agglomerating at the surface as their concentration is increased. Indeed, it is known that materials with high surface roughness exhibit a decreased wettability [63].

### 3.6. Electrical Properties of PNIPAM/MWCNT-COOH Hydrogels

**Figure 10:**
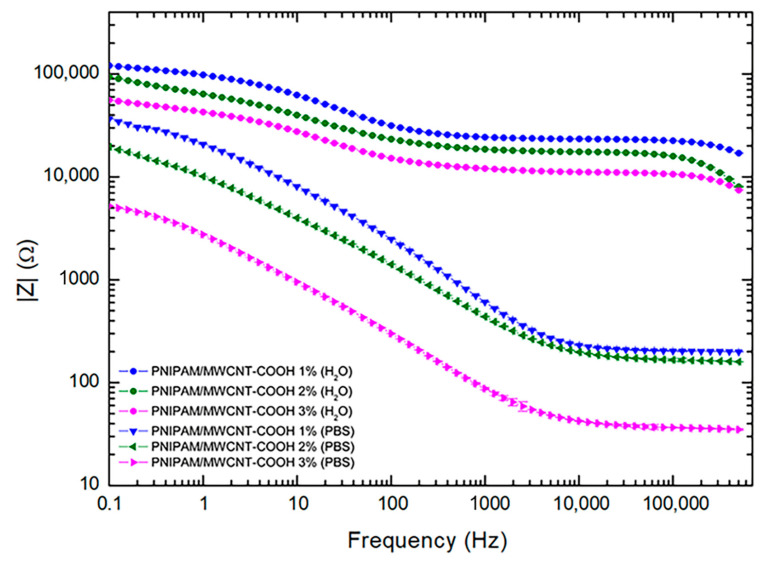
Log-log plot of the impedance modulus of PNIPAM/MWCNT-COOH composite hydrogels with 1, 2, and 3 wt% of filler hydrated in pure water (circles) and in PBS solution (squares). Data points represent mean values (n = 10) ± standard deviation. Where not visible error bars are smaller than symbols.

The electrical properties of the composite hydrogels with 1, 2, and 3 wt% of carboxyl-functionalized carbon nanotubes were investigated by electrical impedance spectroscopy. Figure 10 shows the impedance modulus of the hydrogels swollen in pure water and in PBS solution in the frequency range 0.1 Hz–500 kHz. Within this frequency range, composite hydrogels showed two different behaviors: a frequency-dependent response at low frequencies and a frequency-independent behavior at high frequencies. At low frequencies, there is a dominance of capacitive pathways in the conduction mechanism, while at high frequencies, the resistive networks of the MWCNT-COOH resulting from tunneling, which dominate the conduction mechanism and thus the impedance response, remain constant. Within this frequency range, in both cases, pure water and PBS swelling solutions, a trend is clearly observed correlating with the number of added nanotubes. Upon increasing the MWCNT-COOH content, the impedance value of the composite hydrogels decreases. For this study, hydrogels with 3 wt% of MWCNT-COOH were the most conductive. When comparing the results in pure water and in PBS solution, it was found that the impedance values of hydrogels hydrated in water are much higher than those obtained in PBS solution. This result was expected and is ascribed to the salt ion content of the PBS solution which contributes to the conductivity of the composite hydrogels.

**Figure 11:**
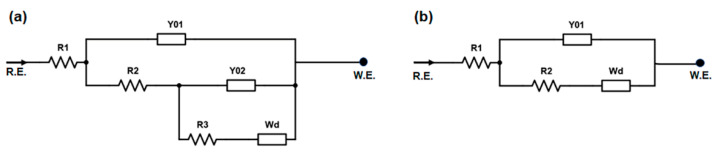
Equivalent circuit models used for fitting the electrical impedance data of hydrogel samples hydrated in (**a**) water and (**b**) PBS solution.

The impedance spectroscopy data were fitted using the equivalent circuit models shown in Figure 11. In particular, two different models were found to best fit the data obtained in the presence of water or PBS solution as swelling liquid. For hydrogels swollen in PBS solution, a Randles equivalent circuit model with a Warburg element (Wd), which describes the mass transport resistance, was used (Figure 11b). The cell ohmic resistance R_1_ corresponds to the resistance associated with the ionic charge transport through the hydrogel, whereas the charge transport resistance R_2_ represents the resistance associated with the electrical kinetics. For hydrogels hydrated in pure water, the equivalent circuit that best fitted the impedance data was a Randles cell with an additional subcircuit of Randles type (Figure 11a).

**Figure 12:**
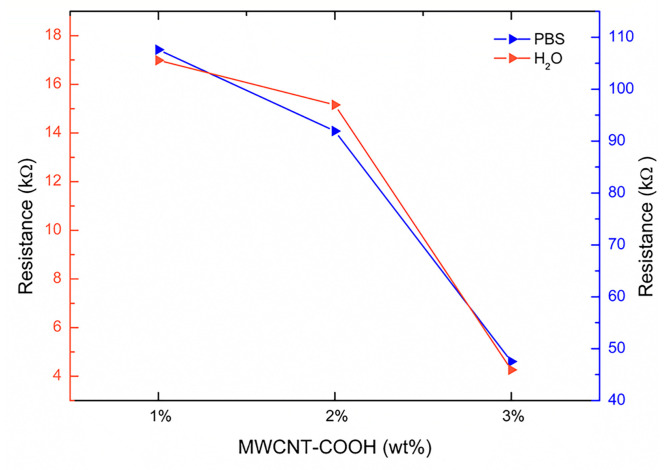
Electrical resistance of PNIPAM/MWCNT-COOH composite hydrogels hydrated in pure water (right axis) and in PBS solution (left axis) as a function of filler concentration. Resistance values corresponding to R_2_ values determined from fitting of impedance data.

The mean values of the equivalent circuit parameters used for fitting the impedance data of the PNIPAM/MWCNT-COOH hydrogels in water and in PBS are summarized in Tables S2 and S3, respectively. Figure 12 reports the equivalent resistances R_2_, representing the charge transport resistances, of the hydrogels with 1, 2, and 3 wt% of nanotubes determined by the impedance data fitting with the equivalent circuit models of Figure 11. The trend, for both cases, water and PBS solution, is the same but the resistance values of the hydrogels swollen in PBS solution are significantly lower, with an order of magnitude difference (Figure 12). The lowest resistance value achieved in this study, across the 1-mm thick sample (1 cm × 1 cm), was approximately 4 kΩ for the hydrogels with 3 wt% of MWCNT-COOH. These results outline the effective role of carboxylated carbon nanotubes in enhancing the electrical conductiviy of the composite hydrogels and provide new directions for future research, which should aim at the evaluation of the hydrogel electrical properties at nanotube concentrations above 3 wt%.

## 4. Conclusions

In this study, PNIPAM-based composite hydrogels embedding carboxyl-functionalized multi-walled nanotubes (MWCNT-COOH) were synthesized using a two-step photopolymerization approach. Swelling tests in water and in PBS solution showed the hydrogel capacity to absorb and retain large amounts of water, with values higher than 90% in both cases. This result makes these hydrogels excellent candidates for the simulation of specific aspects of tissue microenvironments and extracellular matrix (ECM). Contact angle measurements confirmed the high degree of surface hydrophilicity of the hydrogels, with values coherently increasing at high concentrations of the carbon nanotube filler. Degradation tests at physiological conditions showed a minimal degradation of the hydrogels (~0.30%) over four weeks. The potential release of the MWCNT-COOH filler is further inhibited by electrostatic interactions between the negatively charged nanotubes and the positively charged PNIPAM network. The thermal response of the PNIPAM-based hydrogels was investigated by calorimetry (DSC). When increasing the concentration of MWCNT-COOH, the glass transition temperature decreases, indicating that the nanotubes inhibit polymer cross-linking by steric hindrance. Associated with the decrease of glass transition temperature, a decrease in heat capacity was also observed, indicating a favorable interaction of the carboxyl-functionalized nanotubes with the PNIPAM polymer matrix. The temperature-responsive behavior of the hydrogels was analyzed in pure water and in PBS solution. In both cases, an increase of the nanotube content leads to a shift of the volume phase transition to higher temperatures due to higher hydrogen bond density. When the hydrogels are swollen in PBS solution, the transition occurs at lower temperatures due to a screening effect by the salt ions, which leads to a partial neutralization of the positive charges on the hydrogel network. In the end, EIS was used to assess the electrical conductivity of the composite hydrogels in PBS solution. The analysis revealed a consistent behavior with the hydrogels at higher concentrations of MWCNT-COOH having lower impedance values over the frequency range 0.1 Hz–500 kHz. Overall, the two-step photopolymerization approach enables the possibility of a clinical application of these hydrogels in situ. In fact, the initial steps consisting in the nanotube dispersion in the PNIPAM gel obtained by the first photopolymerization can be carried out beforehand. Next, the second photopolymerization could be performed in situ after the application of the viscous PNIPAM/MWCNT-COOH gel to the treatment site.

## Figures and Tables

**Table 1:** Residue values (%) over four weeks evaluated from the supernatant liquid above PNIPAM/MWCNT-COOH hydrogels with 3 wt% of carbon nanotubes.

Time (Week)	Residue (%)
1	0.17 ± 0.02
2	0.20 ± 0.04
3	0.24 ± 0.02
4	0.30 ± 0.03

**Table 2:** Comparison of volume phase transition temperatures (VPTT) and related enthalpy variations (ΔH) of PNIPAM/MWCNT-COOH hydrogels equilibrated in pure water and in PBS solution.

Hydrogel Sample	VPTT in H_2_O [°C]	VPTT in PBS [°C]	ΔH_H_2_O_ [J/g]	ΔH_PBS_ [J/g]
PNIPAM	37.15 ± 0.10	33.97 ± 0.16	0.65 ± 0.05	0.95 ± 0.13
PNIPAM/MWCNT-COOH 0.5%	37.49 ± 0.06	34.43 ± 0.08	0.46 ± 0.19	1.32 ± 0.12
PNIPAM/MWCNT-COOH 1%	38.97 ± 0.20	34.73 ± 0.09	1.06 ± 0.06	1.00 ± 0.06
PNIPAM/MWCNT-COOH 2%	38.05 ± 0.12	35.17 ± 0.12	0.53 ± 0.04	0.47 ± 0.07
PNIPAM/MWCNT-COOH 3%	38.49 ± 0.22	35.39 ± 0.11	0.46 ± 0.05	0.29 ± 0.03

## Data Availability

Data is contained within the article or Supplementary Material.

## Supplementary Materials

The following supporting information can be downloaded at: https://www.mdpi.com/article/10.3390/polym15041022/s1, Figure S1: Particle size distribution of MWCNT-COOH aggregates after dispersion in the PNIPAM gel obtained by the first photopolymerization. Concentration of nanotubes equal to (a) 0.5 wt%, (b) 1.0 wt%, (c) 2.0 wt%, (d) 3.0 wt%; Figure S2: Contact angle analysis at 3 different time frames for pure PNIPAM hydrogel and PNIPAM/MWCNT-COOH composites with different nanotube concentrations (0.5, 1, 2, and 3 wt%); Figure S3: Images of PNIPAM and PNIPAM/MWCNT-COOH composite hydrogels during the two-step photopolymerization process. Top: PNIPAM/MWCNT-COOH gels after dispersion of the nanotubes in the PNIPAM gel. Bottom: top surface of the final hydrogels after the second step of photopolymerization and purification in water; Table S1: Mean particle size of MWCNT-COOH aggregates after dispersion in the PNIPAM gel obtained by the first photopolymerization; Table S2: Mean values of the parameters of the equivalent circuit (Figure 11a) used for fitting the electrical impedance data of hydrogels in water; Table S3: Mean values of the parameters of the equivalent circuit (Figure 11b) used for fitting the electrical impedance data of hydrogels in PBS.
