# Supplementary material for: Conductive and Thermo-Responsive Composite Hydrogels with Poly(N-isopropylacrylamide) and Carbon Nanotubes Fabricated by Two-Step Photopolymerization
Source: Polymers (Basel). 2023 Feb 18;15(4):1022. doi: 10.3390/polym15041022 (PMC9962410; doi:10.3390/polym15041022)
Supplement: Supplementary file 1 [file polymers-15-01022-s001.zip › polymers-2181780-supplementary.pdf]

Supplementary Materials

Conductive and Thermo-Responsive Composite Hydrogels with Poly(N-Isopropylacrylamide) and Carbon Nanotubes Fabricated by Two-Step Photopolymerization

Gianluca Ciarleglio, Elisa Toto and Maria Gabriella Santonicola*

Department of Chemical Engineering Materials Environment, Sapienza University of Rome, Via del Castro Laurenziano 7, 00161 Rome, Italy

*Correspondence: mariagabriella.santonicola@uniroma1.it; Tel.: +39-06-49766372

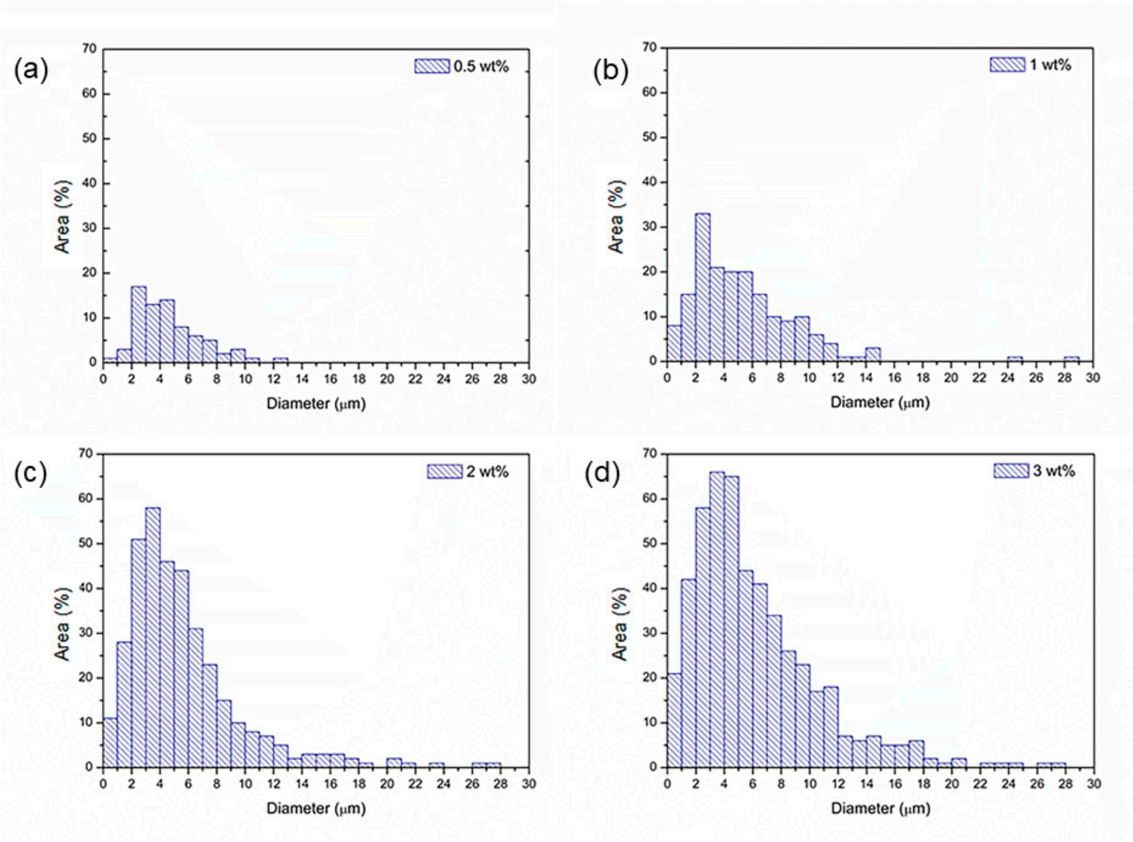

Figure S1. Particle size distribution of MWCNT-COOH aggregates after dispersion in the PNIPAM gel obtained by the first photopolymerization. Concentration of nanotubes equal to (a) 0.5 wt%, (b) 1.0 wt%, (c) 2.0 wt%, (d) 3.0 wt%.

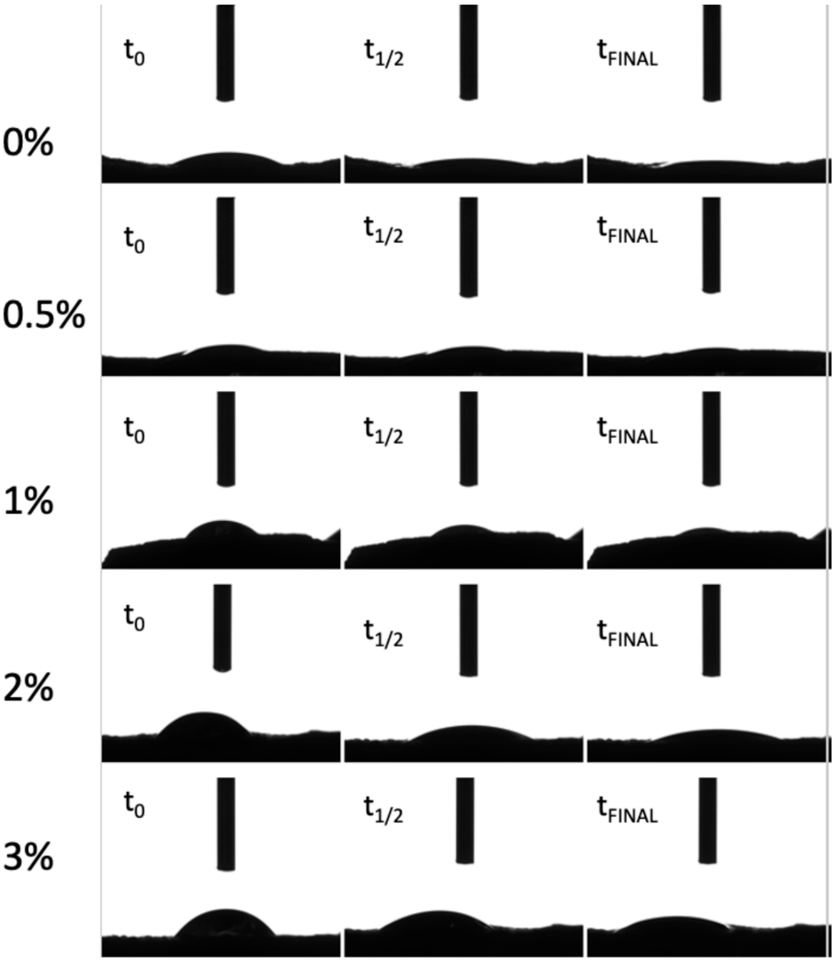

Figure S2. Contact angle analysis at 3 different time frames for pure PNIPAM hydrogel and PNIPAM/MWCNT-COOH composites with different nanotube concentrations (0.5, 1, 2, and 3 wt%).

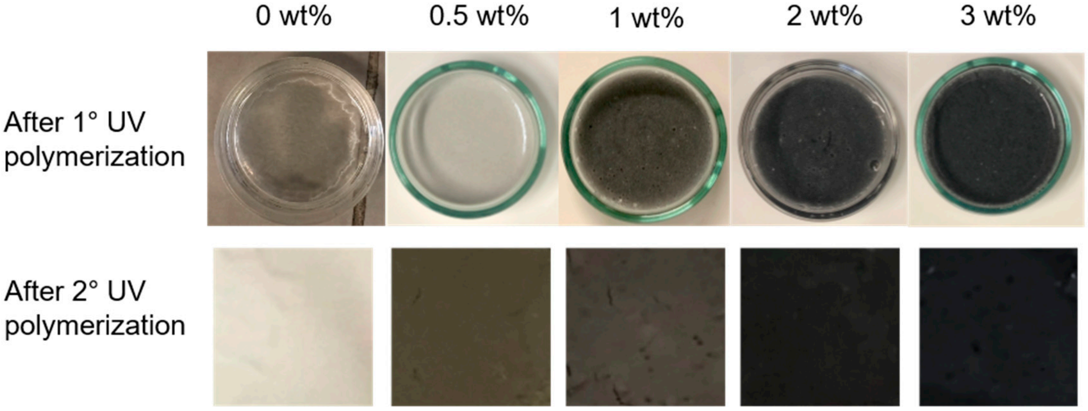

Figure S3. Images of PNIPAM and PNIPAM/MWCNT-COOH composite hydrogels during the two-step photopolymerization process. Top: PNIPAM/MWCNT-COOH gels after dispersion of the nanotubes in the PNIPAM gel. Bottom: top surface of the final hydrogels after the second step of photopolymerization and purification in water.

Table S1. Mean particle size of MWCNT-COOH aggregates after dispersion in the PNIPAM gel obtained by the first photopolymerization.

MWCNT-COOH (wt%)	Mean Particle Size (μm)
0.5	4.58 ± 2.24
1	5.15 ± 3.13
2	5.43 ± 3.62
3	5.96 ± 3.92

Table S2. Mean values of the parameters of the equivalent circuit (Figure 11a) used for fitting the electrical impedance data of hydrogels in water.

MWCNT-COOH (wt%)	R_1 [Ω]	R_2 [Ω]	Y01 [$\alpha\times\Omega^{-1}\times s^{\alpha}$]	α_{01}	Wd [$n\times\Omega^{-1}\times s^{0.5}$]	Goodness of Fit
1	$2.1\times10^{2} \pm 9.1$	$1.7\times10^{4} \pm 2.9\times10^{3}$	$5.0\times10^{-6} \pm 4.2\times10^{-7}$	$6.8\times10^{-1} \pm 7.3\times10^{-3}$	$5.1\times10^{-5} \pm 9.1\times10^{-6}$	$1.4\times10^{-2} \pm 2.4\times10^{-4}$
2	$1.5\times10^{2} \pm 3.3$	$1.4\times10^{4} \pm 1.5\times10^{3}$	$1.5\times10^{-5} \pm 1.4\times10^{-6}$	$6.0\times10^{-1} \pm 9.5\times10^{-3}$	$1.5\times10^{-4} \pm 1.8\times10^{-5}$	$1.0\times10^{-2} \pm 5.6\times10^{-4}$
3	$3.5\times10^{1} \pm 7.9\ 10^{-1}$	$4.6\times10^{3} \pm 4.6\times10^{2}$	$6.1\times10^{-5} \pm 7.3\times10^{-6}$	$6.2\times10^{-1} \pm 9.8\times10^{-3}$	$4.9\times10^{-4} \pm 1.7\times10^{-4}$	$9.6\times10^{-3} \pm 6.8\times10^{-4}$

MWCNT-COOH (wt%)	Y_{01} [$\alpha\times\Omega^{-1}\times s^{\alpha}$]	α_{01}	Y_{02} [$\alpha\times\Omega^{-1}\times s^{\alpha}$]	α_{02}
1	$5.8\times10^{-10} \pm 1.1\times10^{-10}$	$8.3\times10^{-1} \pm 1.2\times10^{-1}$	$9.0\times10^{-7} \pm 9.1\times10^{-8}$	$6.9\times10^{-1} \pm 1.9\times10^{-2}$
2	$2.7\times10^{-7} \pm 1.3\times10^{-7}$	$3.2\times10^{-1} \pm 1.1\times10^{-1}$	$1.5\times10^{-6} \pm 4.9\times10^{-7}$	$5.8\times10^{-1} \pm 2.0\times10^{-1}$
3	$6.1\times10^{-9} \pm 5.6\times10^{-10}$	$6.18\times10^{-1} \pm 1.3\times10^{-1}$	$2.5\times10^{-6} \pm 4.9\times10^{-7}$	$6.7\times10^{-1} \pm 2.3\times10^{-2}$

Table S3. Mean values of the parameters of the equivalent circuit (Figure 11b) used for fitting the electrical impedance data of hydrogels in PBS.

MWCNT-COOH (wt%)	R1 [Ω]	R2 [Ω]	R3 [Ω]	Wd [$n\times\Omega^{-1}\times s^{0.5}$]	Goodness of Fit
1	$4.9\times10^{3} \pm 3.2\times10^{3}$	$2.0\times10^{4} \pm 5.9\times10^{3}$	$8.6\times10^{4} \pm 3.5\times10^{3}$	$6.6\times10^{-5} \pm 1.3\times10^{-5}$	$5.1\cdot10^{-4} \pm 6.2\times10^{-5}$
2	$5.2\times10^{2} \pm 6.0\times10$	$1.8\times10^{4} \pm 5.5\times10^{3}$	$7.9\times10^{4} \pm 2.7\times10^{4}$	$2.8\times10^{-5} \pm 9.2\times10^{-6}$	$9.1\cdot10^{-3} \pm 6.2\times10^{-4}$
3	$6.5\times10^{2} \pm 1.3\times10^{3}$	$1.1\times10^{4} \pm 2.6\times10^{3}$	$3.5\times10^{4} \pm 2.5\times10^{3}$	$8.8\times10^{-5} \pm 7.4\times10^{-5}$	$1.3\cdot10^{-3} \pm 6.2\times10^{-4}$
